# Assessing the quality and integrating the evidence and strength of recommendations in the guidelines for gastric precancerous lesions

**DOI:** 10.1186/s12885-025-13687-y

**Published:** 2025-02-15

**Authors:** Jia-yin Ou, Yang Liu, Lang Zhang, Tian-qi Luo, Jia-yu Li, Li-ming Lu, Lin Wang, Qiu-rong He, Xin Liu, Hua-feng Pan

**Affiliations:** 1 Science and Technology Innovation Center, Guangzhou University of Chinese Medicine, Guangzhou, Guangdong Province 510405 China; 2 The Second Clinical Medical College of Guangzhou University of Chinese Medicine, Guangzhou, Guangdong Province 510405 China; 3 The First Clinical Medical College of Guangzhou University of Chinese Medicine, Guangzhou, Guangdong Province 510405 China; 4 South China Research Center for Acupuncture and Moxibustion, Guangzhou University of Chinese Medicine, Guangzhou, Guangdong Province 510006 China; 5 Medical College of Acu-Moxi and Rehabilitation, Guangzhou University of Chinese Medicine, Guangzhou, Guangdong Province 510006 China; 6 School of Pharmaceutical Sciences, Guangzhou University of Chinese Medicine, Guangzhou, Guangdong Province 510006 China

**Keywords:** Clinical practice guidelines, AGREE II, RIGHT, AGREE-REX, Gastric precancerous lesions, Quality appraisal

## Abstract

**Background:**

Clinical practice guidelines (CPGs) are intended to offer appropriate recommendations for clinical practice based on the available evidence while acknowledging existing gaps and uncertainties. The quality of CPGs for gastric precancerous lesions (GPL), distribution of evidence quality, and strength of recommendations are unknown.

**Objective:**

Systematically evaluate the quality of CPGs for GPL and identify areas for improvement in the development process.

**Methods:**

PubMed, Embase, Cochrane Library, Cumulative Index to Nursing and Allied Health Literature, and six online CPG repositories were systematically searched for CPGs related to GPL. Three researchers independently assessed the methodological quality of the included CPGs by using the AGREE II tool. The reporting and recommendation quality of the CPGs were evaluated using the RIGHT and AGREE-REX tools through consensus. Evidence-based CPGs were analyzed using the Grading of Recommendation Assessment, Development, and Evaluation system to determine the distribution of quality of evidence and strength of recommendations.

**Results:**

A total of 4046 records were identified; nine CPGs met the eligibility criteria for this study. The mean overall score for the methodological quality of the CPGs was 46.22%. Among the six domains, the mean score for clarity of presentation was the highest (71.67%), while the mean score for applicability was the lowest (24.56%). Among the nine CPGs, only one was considered high quality. Regarding reporting quality, domains 1, 3, and 4 had mean reporting rates equal to or higher than 60%. The mean overall score for the recommendation quality was 19.11%. In total, 235 recommendations were identified through the screening process, of which 64.4% were classified as strong. However, only 17.5% of the strong recommendations were supported by high-quality evidence.

**Conclusion:**

The overall quality of CPGs for GPL was poor, with uneven quality across domains. In addition, the consistency between the strength of recommendations and the quality of evidence was poor.

**Supplementary Information:**

The online version contains supplementary material available at 10.1186/s12885-025-13687-y.

## Introduction

Gastric cancer remains a significant global issue and is the fifth most prevalent cancer and the third leading cause of cancer-related deaths, according to the latest published international cancer statistics [1]. Despite their potential for early recognition and treatment, most gastric cancer cases are diagnosed at advanced stages, resulting in high mortality among patients. However, in the early development of gastric cancer, there are often some reversible early precancerous lesions, including atrophic gastritis (AG), intestinal metaplasia (IM), and dysplasia, which can be used as markers for the early development of gastric cancer [2, 3]. The probabilities of atrophic gastritis, intestinal metaplasia, and moderate-to-mild dysplasia developing into gastric cancer within 5 years were 0.1%, 0.25%, and 0.6%, respectively, and that of severe dysplasia was as high as 6% [4].

Clinical practice guidelines (CPGs) for gastric precancerous lesions (GPL) can assist doctors in systematically assessing the risk and progression of GPL and in performing interventions based on risk stratification to reduce the occurrence of gastric cancer in the future [5]. Therefore, several countries and international organizations have constructed and revised CPGs for GPL to enhance its diagnostic and interventional efficacy. CPGs are established through systematic evaluations of evidence and provide recommendations that consider the advantages and disadvantages of various interventions, with the ultimate aim of providing patients with optimal healthcare and aiding medical practitioners in making well-informed clinical decisions [6]. The efficacy of CPGs is contingent on several pivotal factors, including their quality, rigor of the methodology employed, and transparency of the development process [7]. Notably, superior-quality CPGs correlate with more favorable treatment outcomes by by providing recommendations such as appropriate clinical treatment and management [8]. In addition, a clear definition of the level of evidence on which a CPG’s recommendations are based is also crucial [9, 10]. Previous studies have demonstrated considerable heterogeneity in CPG quality across various domains [11]. Common issues encountered in CPGs include an overwhelming volume of complex documentation, limited clarity regarding supporting evidence, disregard for important stakeholders, absence of editorial independence, and inadequate applicability [11–13]. Nevertheless, there remains a lack of quality assessments of existing CPGs for GPL, as well as a lack of comprehensive elucidation of the level of evidence underpinning these CPGs.

The Appraisal of Guidelines for Research and Evaluation II (AGREE II) is a valuable and dependable instrument for assessing the methodological quality of CPGs [14–16]. AGREEII can be used to evaluate CPGs for various diseases [14, 17–20]. However, a trustworthy CPG should meet rigorous methodological criteria and have transparent, clear, and complete reporting [21]. The Reporting Items for Practice Guidelines in Healthcare (RIGHT) has been widely implemented as a CPG reporting standard and is a useful tool for CPG makers in clinical medicine and public health and for journal editors, peer reviewers, and CPG users [22, 23]. To enhance the quality of CPG recommendations and guarantee their credibility, reliability, and implementation in clinical practice, an international research group devised a supplementary system called the Appraisal of Guidelines Research and Evaluation Recommendations Excellence (AGREE-REX). This system serves as an extension of the AGREE II [18, 24].

Therefore, this study aimed to use three evaluation tools (AGREE II, RIGHT, and AGREE-REX) to evaluate the methodology, reporting, and recommendation quality of CPGs for GPL and analyze the distribution of the quality of evidence and strength of recommendations in these CPGs. The purpose was also to identify potential factors that contribute to the substandard quality of CPGs and to emphasize potential avenues for improvement, thereby offering a reliable reference for the future development of CPGs in the field of GPL.

## Methods

### Eligibility criteria

CPGs were included if they: (1) focused on the diagnosis and management of GPL, which includes AG, IM, and dysplasia [4, 25]; (2) were written in English; and (3) were published from January 2011 to April 2023. Consistent with the methods used in previous studies [17, 20], both evidence- and consensus-based CPGs were included. For the updated CPG, only the most recent version was considered.

CPGs were excluded if: (1) full texts were not available; (2) they were reviews, letters, editorials, and correspondence studies; and (3) they were interpreted, translated, or adapted, as well as duplicate publications.

### Search strategy

A researcher conducted a literature search, and four databases (PubMed, Embase, Cochrane Library, Cumulative Index to Nursing and Allied Health Literature) were systematically searched along with six CPG repositories: the National Institute for Health and Clinical Excellence, Scottish Intercollegiate Guidelines Network, Guidelines International Networks, Agency for Healthcare Research and Quality, National Health and Medical Research Council, and World Health Organization. These databases were searched by combining keywords and medical subject headings for CPGs and GPL; detailed search strategies are provided in Supplementary File 1. The search period was from January 1, 2011, to April 1, 2023.

### Study selection

The retrieved records were exported to EndNote X7.7.1 (Thomson Reuters Corporation, CA, USA), and duplicates were eliminated using the software command “Find duplicates” and manual inspection. Two researchers screened the remaining records based on the titles and abstracts of the relevant articles. Subsequently, the researchers obtained the full texts of potentially relevant CPGs for further analysis, and the full texts of the included studies were assessed using the same inclusion and exclusion criteria. When a discrepancy arose regarding the assessment of CPGs by the two researchers, deliberations were held with the involvement of another researcher to achieve a consensus.

### Data extraction

Two researchers performed data extraction and retrieved all supplementary materials related to the methodology for developing CPGs for a more comprehensive assessment of the included CPGs, and another researcher checked consistency. The characteristics of each CPG were extracted to understand their basic information for further subgroup analyses. Using the author and publication year as CPG IDs, we extracted the following data: country, type of development organization (medical association or society / expert panel), use of the CPG quality tool (yes, no, or not stated), version (updated or first time), development method (evidence-based (EB) or consensus-based (CB)), and source of funding (yes, no, or not stated). When a CPG was identified as not being developed by a specific association, it was classified by an “society / expert panel.”

### Quality evaluation

The quality of the included CPGs was systematically evaluated using AGREE II, RIGHT, and AGREE-REX tools. To prepare for the evaluation, each assessor underwent training on the recommended training modules on the AGREE and RIGHT websites and learned how to evaluate each item of the CPGs in the AGREE II, RIGHT, and AGREE-REX user manuals. Subsequently, three CPGs were chosen for pilot evaluation, and discussions were held to resolve any notable disparities in scores between assessors and to clarify any other aspects that could enhance assessors’ evaluative skills.

#### Evaluation of methodological quality

Three researchers independently evaluated the methodological quality of the CPGs using the AGREE II tool, which consists of 23 items grouped into six domains: scope and purpose, stakeholder involvement, rigor of development, clarity of presentation, applicability, and editorial independence [26]. The researchers assessed each AGREE II item of the CPG according to the AGREE II guidelines and the criteria suggested for each item and scored it using a 7-point Likert scale [14], which ranged from 1 (strongly disagree) to 7 (strongly agree), where scores between 2 and 6 indicated inadequate adherence to the AGREE II item criteria. Three researchers independently evaluated and recorded the scores, after which the AGREE II score of each researcher was collated and recorded in a Microsoft Excel spreadsheet by one researcher. When there was a difference of more than two points in the score of any item included in the CPG, it was reassessed or discussed in a meeting until the score difference was reduced or consensus was reached. The individual domain scores for each CPG were then compiled and calculated as a percentage of the maximum attainable score, employing the formula (score obtained—lowest possible score)/(highest score—lowest possible score) × 100% [27].

In the comprehensive assessment of CPG, the overall rating item was evaluated using a 7-point Likert scale and calculated as percentages, employing the same methodology utilized in prior studies [18, 28]. Regarding the second global evaluation item, a CPG was deemed to be of high quality, consistent with previous research [18, 29, 30], if all three domains in the AGREE II tool were considered the most important: stakeholder engagement (domain 2), rigor of development (domain 3), and editorial independence (domain 6), attaining a minimum of 50% of the maximum possible score.

#### Evaluation of report quality

The RIGHT statement assesses the reporting quality of CPGs. It includes seven domains: basic information, background, evidence, recommendations, review and quality assurance, funding, declaration of interests and management, and other information. The seven domains involved 22 items accompanied by elaboration documents [31]. Each item’s reporting quality was assessed using a three-level scale by three researchers through discussion, with ratings of “completely reported,” “partially reported,” and “not reported” corresponding to numerical values of 1, 0.5, and 0 [32, 33], respectively. The reporting rate for each domain in each CPG was then calculated based on (scores from items “reported” in each domain)/(the total number of items in each domain) × 100%, and the overall reporting rate of each CPG was calculated based on the score for the “reported” item for all domains/35 [27].

#### Evaluation of recommendation quality

The AGREE-REX tool was designed to provide guidance for reporting recommendations for CPGs [24]. It encompasses three essential quality domains comprising nine individual items: clinical applicability, values and preferences, and implementability. Three researchers evaluated the recommendation quality of the included CPGs using the AGREE-REX instrument through in-person discussions, assigning item scores ranging from 1 to 7. The domain scores were calculated following the same methodology described in AGREE II.

### Quality of evidence and strength of recommendations

We first identified the grading system used for each evidence-based CPG and the evidence of different qualities and recommendations for different strengths. The Grading of Recommendation Assessment, Development, and Evaluation (GRADE) system [34, 35], which is widely recognized by many professional societies, has been adopted as the most ideal and commonly used method for evidence grading and recommendation designation. To achieve a high degree of standardization of the statistical results, graded evidence and recommendations from the CPGs were incorporated into the GRADE system whenever possible.

During the evaluation process, recommendations lacking clarity regarding the quality of evidence and strength were excluded. In cases where multiple qualities of evidence were present to support a recommendation, the evidence with the highest quality was prioritized. By re-evaluating each CPG, we determined the distribution of evidence quality and recommendation strength in evidence-based CPGs.

### Statistical analysis

The AGREE II, RIGHT, and AGREE-REX scores of the researchers were entered into Microsoft Excel (Microsoft, WA, United States) to compute standardized scores for each domain and the overall scores for each CPG. Continuous variables were presented as means ± standard deviation (SD), while categorical variables were presented as frequencies and percentages. Spearman’s correlation was used to investigate the correlations between the scores of the AGREE II, RIGHT, and AGREE-REX domains. An independent sample t-test was employed to assess the disparities between the two groups, while a one-way analysis of variance was used to compare the differences among multiple groups. We conducted subgroup analyses on the AGREE II, RIGHT, and AGREE-REX domains as well as the overall score, stratified by version (first edition or update), whether funding source is declared, whether conflict of interest is declared, publication country or region, publication time, development method, whether CPG quality assessment tools are used, journal, whether scoring system is used, scope, and disease. The inter-rater reliability of the AGREE II tool was tested using a two-way random-effects model to calculate intra-class correlation coefficients (ICC) and 95% confidence intervals (CI). Consistent with previous studies [17, 20] and according to Landis and Koch [36], the level of inter-rater reliability was categorized as follows: a value between 0.01 and 0.20 indicated a minor degree of agreement, a value between 0.21 and 0.40 denoted a fair degree, a value between 0.41 and 0.60 represented a moderate degree, a value between 0.61 and 0.80 indicated a substantial degree and a value between 0.81 and 1.00 indicated a very good level of agreement. A significance level of *p* < 0.05 was deemed statistically significant. Statistical analyses were performed using SPSS version 27 (IBM SPSS Statistics, IBM Corporation), and the bioinformatics online service platform (http://www.bioinformatics.com.cn/) was used to generate Spearman’s correlation coefficient maps for the scores of each CPG evaluation tool.

## Results

### Literature screening

**Fig. 1:**
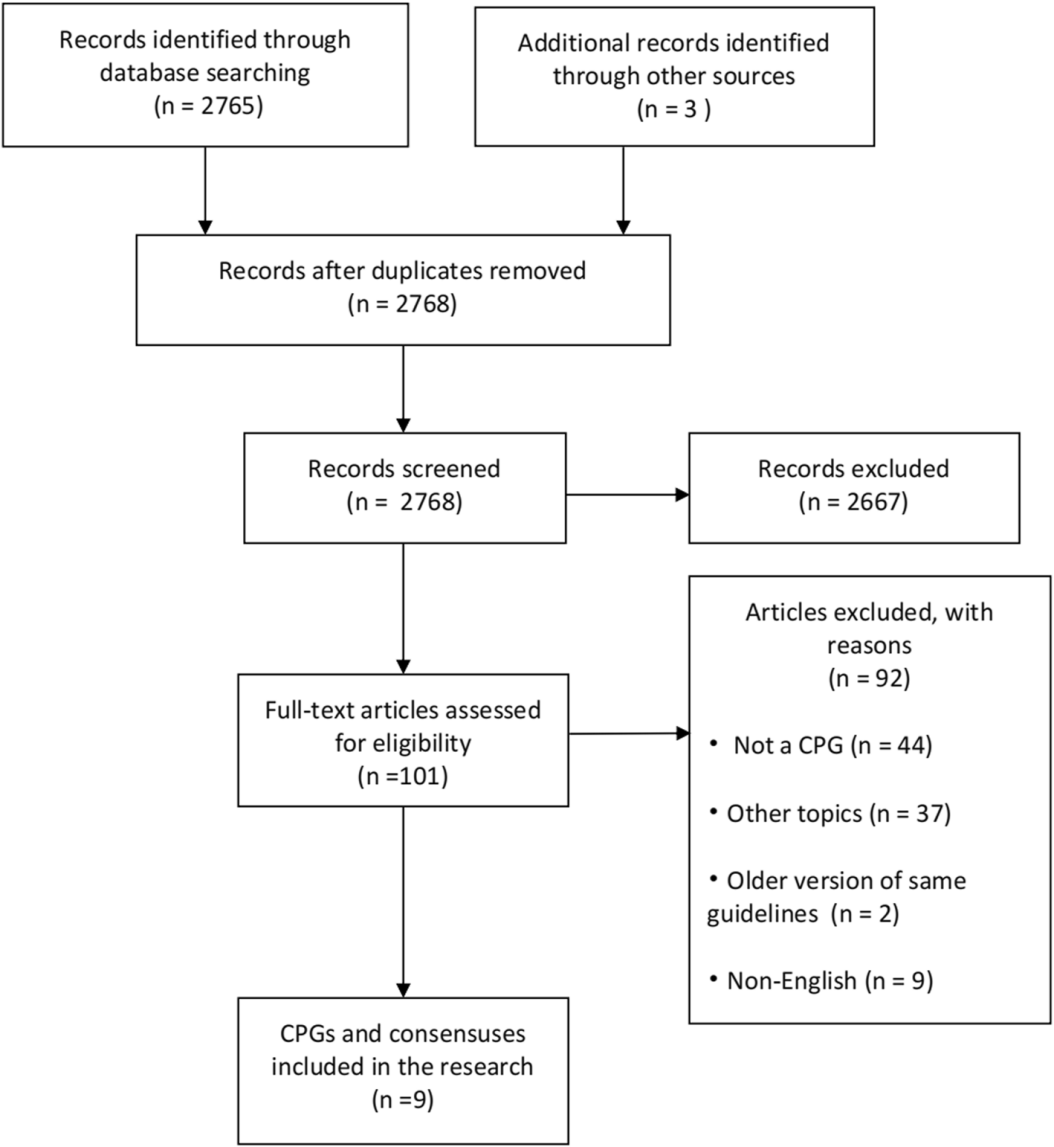
Flow diagram for search and selection of CPGs. CPGs, clinical practice guidelines

A comprehensive search of literature websites yielded 2,765 records supplemented by three additional records from 2 CPG databases, NICE and AHRQ. After the elimination of duplicate records and initial screening, 101 results were obtained. These results were thoroughly analyzed through a full-text literature review and meticulous screening based on the eligibility criteria. Ultimately, nine CPGs were identified (Fig. 1).

### Characteristics of CPGs

Among the nine CPGs identified, the majority originated from the United States (four CPGs), followed by China (two CPGs), Italy, the United Kingdom, and Europe each had one CPG. Notably, one of the CPGs from China was developed by an expert panel, whereas the remaining eight CPGs were developed by medical societies. Of the CPGs, eight were applicable for diagnosis or treatment purposes, two of which were suitable for prevention. Additionally, six CPGs disclosed their funding sources, five declared conflicts of interest, one included methodologists, and one CPG utilized a CPG-quality tool (Table 1).

**Table 1:** Characteristics of included CPGs

CPG ID	Development organization	Type of development organization	Country/region of origin	Development method	Used CPG quality tool	Included CPG methodologist	Journal	Funding sources	Stated conflict of interest
Tang et al., 2012 [37]	CGGDG	society / expert panel	China	EB	No stated	No stated	Chin J Integr Med	Yes	No
Sharaf et al., 2013 [38]	SPCASGE	Medical Association	United States	EB	No stated	No stated	Gastrointest Endosc	Yes	No
Evans et al., 2015 [39]	SPCASGE	Medical Association	United States	EB	No stated	No stated	Gastrointest Endosc	Yes	No
Banks et al., 2019 [40]	BSG	Medical Association	UK	EB	Yes	No stated	Gut	No stated	Yes
Lahner et al., 2019 [41]	SIED, SIGE, SIMI	Medical Association	Italian	EB	No stated	No stated	Dig Liver Dis	No stated	No
Pimentel-Nunes et al., 2019 [4]	ESGE, EHMSG, ESP, SPED	Medical Association	Europe	EB	No stated	No stated	Endoscopy	No stated	Yes
Gupta et al., 2020 [42]	AGA	Medical Association	United States	EB	No stated	Yes	Gastroenterology	Yes	Yes
Shah et al., 2021 [43]	AGA	Medical Association	United States	CB	No stated	No stated	Gastroenterology	Yes	Yes
Wang et al., 2022 [44]	SSDBCATCM, GBCMA	Medical Association	China	EB	No stated	No stated	Chinese Medicine	Yes	Yes

*CPG ID* clinical practice guideline identifier, *EB* evidence-based, *CB* consensus-based, *CGGDG* the Chronic Gastritis Guideline Development Group, *SPCASGE* The Standards of Practice Committee of the American Society for Gastrointestinal Endoscopy (ASGE), *BSG* The British Society of Gastroenterology, *SIED* the Italian Society of Digestive Endoscopy, *SIGE* the Italian Society of Gastroenterology, *SIMI* and the Italian Society of Internal Medicine, *ESGE* European Society of Gastrointestinal Endoscopy, *EHMSG* European Helicobacter, and Microbiota Study Group, *ESP* European Society of Pathology, *SPED* Sociedade Portuguesa de Endoscopia Digestiva, *AGA* American Gastroenterological Association, *SSDBCATCM* The Spleen and Stomach Diseases Branch of the China Association of Traditional Chinese Medicine, *GBCMA* The Gastroenterology Branch of Chinese Medical Association, *UK* United Kingdom of Great Britain and Northern Ireland

### Evaluation of methodological quality

The mean scores of six domains of the AGREE II scale were as follows: domain 1 (scope and purpose) was 64.22% (SD = 16.09%); domain 2 (stakeholder involvement) was 31.78% (SD = 13.77%); domain 3 (rigour of development) was 49.56% (SD = 21.18%); domain 4 (clarity of presentation) was 71.67% (SD = 12.39%), which was the highest score among the six domains; domain 5 (applicability) was 24.56% (SD = 12.10%), which was the lowest among the six domains; domain 6 (editorial independence) was 36.33% (SD = 24.78%) (Table 2). The mean overall score of all CPGs was 46.22% (SD = 9.24%), and only one CPG [40] was considered of high quality. Items 5 “The views and preferences of the target population (patients, public, etc.) have been sought” and item 20 “The potential resource implications of applying the recommendations have been considered” had the lowest scores, both with a mean score below 2, distinguishing them from the other items. Items 12 “There is an explicit link between the recommendations and the supporting evidence” and item 16 “The different options for management of the condition or health issue are clearly presented” had the highest mean scores (Supplementary Table 1).

**Table 2:** Domain and overall scores of CPGs according to AGREE II

CPG ID	Scope and purpose (%)	Stakeholder involvement (%)	Rigour of development (%)	Clarity of presentation (%)	Applicability (%)	Editorial independence (%)	Overall score (%)
Tang et al., 2012 [37]	52	11	88	76	8	0	39
Sharaf et al., 2013 [38]	52	37	36	69	21	25	40
Evans et al., 2015 [39]	70	33	55	78	25	25	47
Banks et al., 2019 [40]	78	59	60	74	47	53	62
Lahner et al., 2019 [41]	50	24	51	69	26	22	40
Pimentel-Nunes et al., 2019 [4]	87	26	64	89	21	50	56
Gupta et al., 2020 [42]	81	43	44	48	11	83	52
Shah et al., 2021 [43]	67	31	13	83	38	50	47
Wang et al., 2022 [44]	41	22	35	59	24	19	33
Mean ± SD	64 ± 16	32 ± 14	50 ± 21	72 ± 12	25 ± 12	36 ± 25	46 ± 9

*CPG ID* clinical practice guideline identifier, *AGREE* Appraisal of Guidelines for Research and Evaluation

The ICC values for each domain were all > 0.80, and the interrater reliabilities of the three researchers were good (Supplementary Table 2). There was a high level of consistency in evaluating the CPGs among the three researchers.

### Evaluation of report quality

The mean overall reporting quality score of CPGs was 54.89% (SD = 14.11%). Among the seven domains of the RIGHT scale, domain 1 (basic information) had the highest mean reporting rate at 70.00% (SD = 21.07%). Conversely, domain 6 (funding and declaration and management of interests) had the lowest mean reporting rate, with a rate of 37.67% (SD = 28.70%). The remaining five domains ranked in descending order of mean scores as follows: domain 3 (evidence) was 67.78% (SD = 16.42%), domain 4 (recommendations) was 60.33% (SD = 12.55%), domain 7 (other information) was 57.44% (SD = 22.15%), domain 5 (review and quality assurance) was 55.56% (SD = 27.32%), and domain 2 (background) was 52.22% (SD = 17.04%) (Table 3). Among all the items, item 1b “Describe the year of publication of the guideline” achieved the highest mean reporting results, with a rate of 100% (SD = 0%). Conversely, item 8b “Describe the setting(s) for which the guideline is intended, such as primary care,low- and middle-income countries, or inpatient facilities” achieved the poorest mean reporting rate of 0% (SD = 0%) (Supplementary Table 3).

**Table 3:** Domain and overall scores of CPGs according to RIGHT

CPG ID	Basic information (%)	Background (%)	Evidence (%)	Recommendations (%)	Review and quality assurance (%)	Funding and declaration and management of Interests (%)	Other information (%)	Overall score (%)
Tang et al., 2012 [37]	83	38	60	50	75	0	17	47
Sharaf et al., 2013 [38]	50	44	50	57	25	13	50	44
Evans et al., 2015 [39]	33	44	70	57	25	25	50	46
Banks et al., 2019 [40]	83	81	80	79	50	75	83	79
Lahner et al., 2019 [41]	83	25	60	50	25	25	67	49
Pimentel-Nunes et al., 2019 [4]	92	69	100	79	100	50	83	80
Gupta et al., 2020 [42]	100	63	60	64	50	25	67	64
Shah et al., 2021 [43]	67	50	50	43	75	38	67	54
Wang et al., 2022 [44]	75	56	80	64	75	88	33	67
Mean ± SD	74 ± 21	52 ± 17	68 ± 16	60 ± 13	56 ± 27	38 ± 29	57 ± 22	59 ± 14

*CPG ID* clinical practice guideline identifier, *RIGHT* Reporting Items for Practice Guidelines in Healthcare

### Evaluation of recommendation quality

The mean overall score for the recommendation quality of the CPGs was 19.11% (SD = 7.74%), with the three domains ranked in descending order of mean scores: clinical applicability, 32.00% (SD = 13.54%); implementability, 24.00% (SD = 7.68%); and values and preferences, 7.00% (SD = 6.80%) (Table 4).

**Table 4:** Domain and overall scores of CPGs according to AGREE-REX

CPG ID	Clinical applicability (%)	Values and differences (%)	Implementability (%)	Overall score (%)
Tang et al., 2012 [37]	61	17	25	33
Sharaf et al., 2013 [38]	11	0	17	7
Evans et al., 2015 [39]	28	0	25	15
Banks et al., 2019 [40]	33	4	25	19
Lahner et al., 2019 [41]	33	4	25	19
Pimentel-Nunes et al., 2019 [4]	39	4	33	22
Gupta et al., 2020 [42]	33	17	33	26
Shah et al., 2021 [43]	22	4	8	11
Wang et al., 2022 [44]	28	13	25	20
Mean ± SD	32 ± 14	7 ± 7	24 ± 8	19 ± 8

*CPG ID* clinical practice guideline identifier, *AGREE-REX* Appraisal of Guidelines for Research and Evaluation-Recommendation Excellence

The three items that obtained the highest mean scores were item 1 “Evidence”, item 2 “Applicability to Target Users”, and item 8 “Purpose”; exclusively, these three items out of the total nine achieved a mean score exceeding three points. The three items with the lowest mean scores pertain to the “values and preferences” domain: item 4 “Values and Preferences of Target Users”, item 6 “Values and Preference of Policy/ Decision Makers”, and item 7 “Values and Preferences of Guideline Developers” (Supplementary Table 4). Remarkably, the mean scores of these three items were exceptionally low, nearly approaching the value of one.

### Subgroup analysis of the scores of AGREE II, RIGHT, and AGREE-REX domains

In the analysis of subgroup scores for each domain and the overall score of the CPG assessment tool, only the domain “editorial independence” (*p* = 0.035) within the “stated conflict of interest” subgroup of AGREE II exhibited a statistically significant difference. There were statistical differences observed in the domain “background” (*p* = 0.009) and “funding and declaration and management of interests” (*p* = 0.027) within the “stated conflict of interest” subgroup of RIGHT. Additionally, statistical differences were found in the “overall score” (*p* = 0.035) within the “year” subgroup of RIGHT. Conversely, the AGREE-REX scores showed no statistical differences in any subgroup (Table 5).

**Table 5:** AGREE II, RIGHT, and AGREE-REX domain and overall scores for different subgroups of CPGs (Mean ± SD, %)

**AGREE II**									
**Subgroups**	**Statistics**	**Scope and purpose**	**Stakeholder involvement**	**Rigour of development**	**Clarity of presentation**	**Applicability**	**Editorial independence**	**AGREE II overall score**	
Version		*P* = 0.759	*P* = 0.499	*P* = 0.356	*P* = 0.251	*P* = 0.621	*P* = 0.974	*P* = 0.901	
First	5(55.56%)	62.6 ± 15.49	34.8 ± 18.31	55.8 ± 20.06	67.2 ± 11.17	22.6 ± 15.47	36.6 ± 32.05	46.60 ± 10.14	
Updated	4(44.44%)	66.25 ± 19	28 ± 4.97	41.75 ± 22.68	77.25 ± 12.97	27 ± 7.53	36 ± 16.35	45.75 ± 9.50	
Founding sources		*P* = 0.36	*P* = 0.52	*P* = 0.416	*P* = 0.366	*P* = 0.26	*P* = 0.678	*P* = 0.149	
Yes	6(66.67%)	60.5 ± 14.68	29.5 ± 11.42	45.17 ± 25.12	68.83 ± 13.17	21.17 ± 10.8	33.67 ± 28.98	43.00 ± 6.70	
No stated	3(33.33%)	71.67 ± 19.3	36.33 ± 19.66	58.33 ± 6.66	77.33 ± 10.41	31.33 ± 13.8	41.67 ± 17.1	52.67 ± 11.37	
Stated conflicts of interest		*P* = 0.186	*P* = 0.312	*P* = 0.347	*P* = 0.794	*P* = 0.345	*P* = 0.035	*P* = 0.186	
Yes	5(55.56%)	70.8 ± 18.17	36.2 ± 14.99	43.2 ± 20.58	70.6 ± 16.95	28.2 ± 14.27	51 ± 22.66	50.00 ± 10.98	
No	4(44.44%)	56 ± 9.38	26.25 ± 11.53	57.5 ± 21.92	73 ± 4.69	20 ± 8.29	18 ± 12.08	41.50 ± 3.70	
Country/region of origin		*P* = 0.618	*P* = 0.448	*P* = 0.115	*P* = 0.670	*P* = 0.872	*P* = 0.341	*P* = 0.942	
America	4(44.44%)	67.50 ± 11.96	36.00 ± 5.29	37.00 ± 17.80	69.50 ± 15.46	23.75 ± 11.18	45.75 ± 27.49	46.00 ± 12.35	
Other countries	5(55.56%)	61.60 ± 19.78	28.40 ± 18.06	59.60 ± 19.40	73.40 ± 10.92	25.20 ± 14.06	28.80 ± 22.40	46.50 ± 4.93	
Year		*P* = 0.449	*P* = 0.499	*P* = 0.344	*P* = 0.678	*P* = 0.278	*P* = 0.091	*P* = 0.367	
> 2017	6(66.67%)	67.33 ± 18.34	34.17 ± 14.30	44.50 ± 18.68	70.33 ± 15.18	27.83 ± 12.80	46.17 ± 23.47	48.33 ± 10.63	
< 2017	3(33.33%)	58.00 ± 10.39	27.00 ± 14.00	59.67 ± 26.31	74.33 ± 4.73	18.00 ± 8.89	16.67 ± 14.43	42.00 ± 4.36	
**RIGHT**									
**Subgroups**	**Statistics**	**Basic information**	**Background**	**Evidence**	**Recommendations**	**Review and quality assurance**	**Funding and declaration and management of Interests**	**Other information**	**RIGHT Overall score**
Version		*P* = 0.391	*P* = 0.718	*P* = 0.264	*P* = 0.936	*P* = 0.215	*P* = 0.265	*P* = 0.93	*P* = 0.620
First	5(55.56%)	79.8 ± 18.21	50.2 ± 21.99	62 ± 10.95	60 ± 12.1	45 ± 20.92	27.6 ± 28.44	56.8 ± 25.12	56.60 ± 14.71
Updated	4(44.44%)	66.75 ± 24.8	54.75 ± 10.69	75 ± 20.82	60.75 ± 14.98	68.75 ± 31.46	50.25 ± 27.16	58.25 ± 21.56	61.75 ± 14.93
Founding sources		*P* = 0.251	*P* = 0.484	*P* = 0.118	*P* = 0.135	*P* = 0.845	*P* = 0.398	*P* = 0.042	*P* = 0.121
Yes	6(66.67%)	68 ± 23.87	49.17 ± 9.13	61.67 ± 11.69	55.83 ± 8.18	54.17 ± 24.58	31.5 ± 30.51	47.33 ± 19.56	53.67 ± 9.81
No stated	3(33.33%)	86 ± 5.2	58.33 ± 29.48	80 ± 20	69.33 ± 16.74	58.33 ± 38.19	50 ± 25	77.67 ± 9.24	59.33 ± 17.62
Stated conflicts of interest		*P* = 0.143	*P* = 0.009	*P* = 0.225	*P* = 0.154	*P* = 0.071	*P* = 0.027	*P* = 0.18	*P* = 0.005
Yes	5(55.56%)	83.4 ± 13.13	63.8 ± 11.99	74 ± 19.49	65.8 ± 14.79	70 ± 20.92	55.2 ± 25.99	66.6 ± 20.42	68.80 ± 10.89
No stated	4(44.44%)	62.25 ± 24.95	37.75 ± 8.96	60 ± 8.17	53.5 ± 4.04	37.5 ± 25	15.75 ± 11.93	46 ± 20.93	46.50 ± 2.08
Country/region of origin		*P* = 0.153	*P* = 0.779	*P* = 0.092	*P* = 0.307	*P* = 0.273	*P* = 0.272	*P* = 0.908	*P* = 0.209
America	4(44.44%)	62.50 ± 28.60	50.25 ± 8.96	57.50 ± 9.57	55.25 ± 8.81	43.75 ± 23.94	25.25 ± 10.21	58.50 ± 9.82	64.40 ± 15.84
Other countries	5(55.56%)	83.20 ± 6.02	53.80 ± 22.67	76.00 ± 16.73	64.40 ± 14.50	65.00 ± 28.50	47.60 ± 35.94	56.60 ± 30.11	52.00 ± 9.09
Year		*P* = 0.051	*P* = 0.224	*P* = 0.348	*P* = 0.373	*P* = 0.311	*P* = 0.056	*P* = 0.072	*P* = 0.035
> 2017	6(66.67%)	83.33 ± 11.74	57.33 ± 19.13	71.67 ± 16.35	63.17 ± 14.72	62.50 ± 26.22	50.17 ± 26.32	66.67 ± 18.26	65.50 ± 12.66
< 2017	3(33.33%)	55.33 ± 25.42	42.00 ± 3.46	60.00 ± 10.00	54.67 ± 4.04	41.67 ± 28.87	12.67 ± 12.50	39.00 ± 19.05	45.67 ± 1.53
**AGREE-REX**									
**Subgroups**	**Statistics**	**Clinical Applicability**		**Values and Preferences**		**Implementability**		**AGREE-REX overall score**	
Version		*P* = 0.619		*P* = 0.527		*P* = 0.692		*P* = 0.501	
First	5(55.56%)	34.20 ± 17.75		8.40 ± 8.02		25.00 ± 5.66		20.80 ± 9.65	
Updated	4(44.44%)	29.25 ± 7.09		5.25 ± 5.50		22.75 ± 10.53		17.00 ± 4.97	
Founding sources		*P* = 0.669		*P* = 0.384		*P* = 0.334		*P* = 0.826	
Yes	6(66.67%)	30.50 ± 16.74		8.50 ± 8.12		22.17 ± 8.59		18.67 ± 9.69	
No stated	3(33.33%)	35.00 ± 3.46		4.00 ± 0.00		27.67 ± 4.62		20.00 ± 1.73	
Stated conflicts of interest		*P* = 0.823		*P* = 0.527		*P* = 0.752		*P* = 0.848	
Yes	5(55.56%)	31.00 ± 6.36		8.40 ± 6.19		24.8 ± 10.21		19.60 ± 5.51	
No stated	4(44.44%)	33.25 ± 20.76		5.25 ± 8.06		23.00 ± 4.00		18.50 ± 10.88	
Country/region of origin		*P* = 0.091		*P* = 0.527		*P* = 0.284		*P* = 0.138	
America	4(44.44%)	23.50 ± 9.47		5.25 ± 8.06		20.75 ± 10.72		22.60 ± 5.94	
Other countries	5(55.56%)	38.80 ± 13.01		8.40 ± 6.19		26.60 ± 3.58		14.75 ± 8.18	
Year		*P* = 0.850		*P* = 0.706		*P* = 0.676		*P* = 0.847	
> 2017	6(66.67%)	31.33 ± 5.75		7.67 ± 5.82		24.83 ± 9.13		19.50 ± 4.93	
< 2017	3(33.33%)	33.33 ± 25.42		5.67 ± 9.82		22.33 ± 4.62		18.33 ± 13.32	

*AGREE* Appraisal of Guidelines for Research and Evaluation, *CPG ID* clinical practice guideline identifier, *AGREE* Appraisal of Guidelines for Research and Evaluation, *RIGHT* Reporting Items for Practice Guidelines in Healthcare, *AGREE-REX* Appraisal of Guidelines for Research and Evaluation Recommendation Excellence

### Correlations among the scores of AGREE II, AGREE-REX, and RIGHT domains

**Fig. 2:**
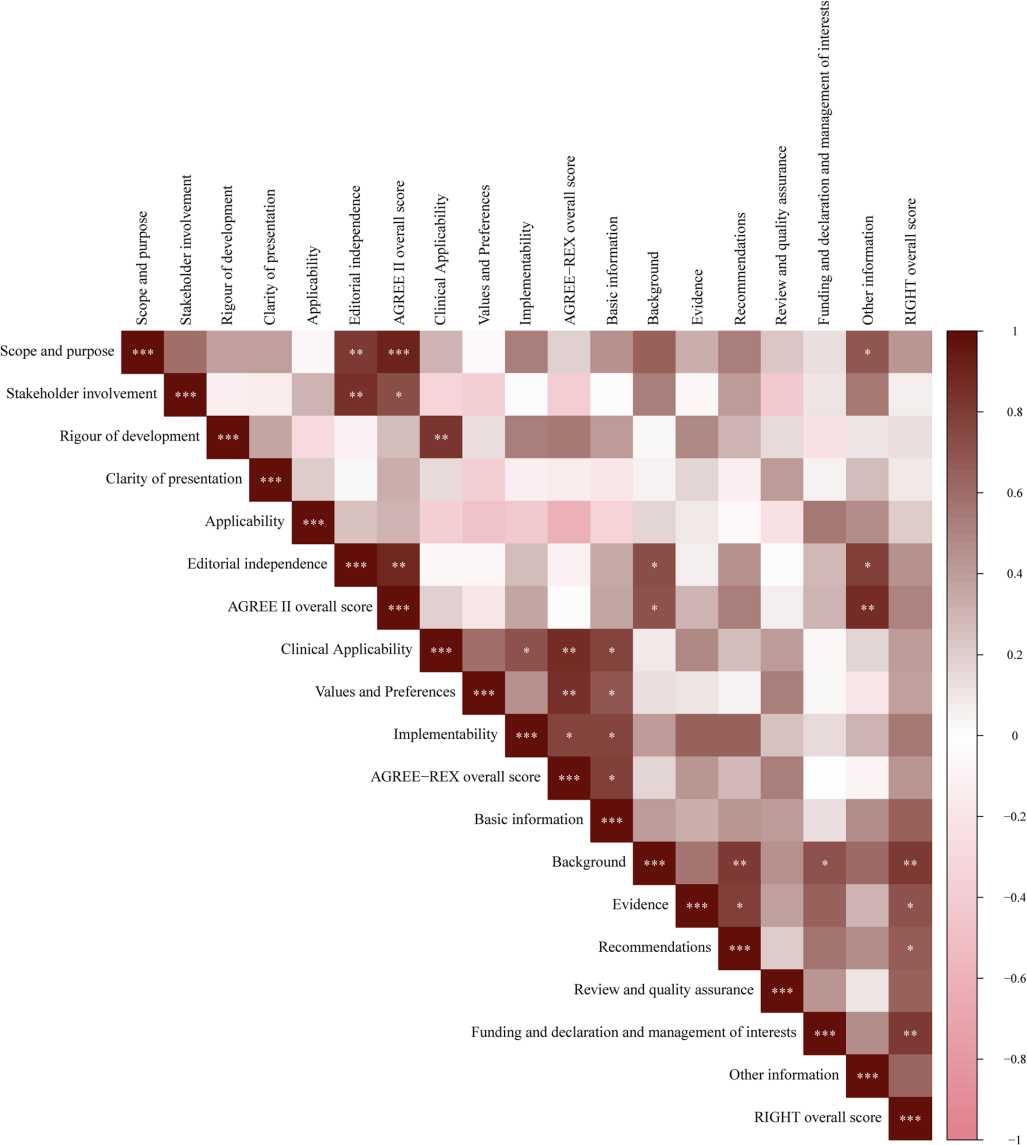
Correlations among the scores of AGREE II, RIGHT, and AGREE-REX domains. AGREE, Appraisal of Guidelines for Research and Evaluation; RIGHT, Reporting Items for Practice Guidelines in Healthcare; AGREE-REX, Appraisal of Guidelines for Research and Evaluation-Recommendation Excellence; *p < 0.05, **p < 0.001, ***p < 0.001; The depth of the color of the squares represents the magnitude of the Spearman correlation coefficient

A partial positive correlation existed between the AGREE II, AGREE-REX, and RIGHT domain scores. Specifically, there was a high positive correlation between “scope and purpose,” “overall score,” and “editorial independence” in AGREE II (*r* > 0.80). Moreover, there is a strong positive correlation between “stakeholder involvement” and “editorial independence” (*r* > 0.80). Furthermore, there was a strong positive correlation between “editorial independence” and the “overall score” and the overall AGREE II score (*r* > 0.80). There was also a positive correlation between the “overall score” of AGREE II and the “other information” in RIGHT (*r* > 0.80). In addition, there were positive correlations (*r* > 0.80) between “background” and “recommendations” of the RIGHT instrument, as well as between “overall score,” “background,” and “funding, declaration, and management of benefits.” In AGREE-REX, positive correlations were found between “overall score,” “clinical applicability,” and “values and preferences” (*r* > 0.80). All correlations were statistically significant (*p* < 0.05) (Fig. 2).

### Quality of evidence and strength of recommendations

Of the eight evidence-based CPGs, seven used the GRADE system for grading evidence, and one used the Oxford Centre for Evidence-based Medicine Levels of Evidence system. In total, 235 recommendations were identified (Table 6).

**Table 6:** The grading system used and the distribution of the quality of evidence and the strength of recommendations among evidence-based CPGs before and after the synthesis of the GRADE system

CPG ID	Name of grading system	Quality of evidence, No. (%)	Strength of recommendation, No. (%)
Tang et al., 2012 [37]	OCEBM Levels of Evidence system	Ia:2 Ib:0 IIa:26 IIb:0 IIIa:0 IIIb:16 IV:11 V:1	A: 4 B: 42 C: 12
Sharaf et al., 2013 [38]	GRADE system	High quality: 1 Moderate quality: 3 Low quality: 28 Very low quality: 1	Strong recommendation: 4 Weak recommendation: 29
Evans et al., 2015 [39]	GRADE system	High quality: 4 Moderate quality: 4 Low quality: 8 Very low quality: 0	Strong recommendation: 8 Weak recommendation: 8
Banks et al., 2019 [40]	GRADE system	High quality: 3 Moderate quality: 9 Low quality: 19	Strong recommendation: 24 High recommendation: 1 Weak recommendation: 6
Lahner et al., 2019 [41]	GRADE system	A: 2 B: 4 C: 5 D: 0	Strong recommendation: 6 Conditional recommendation: 5
Pimentel-Nunes et al., 2019 [4]	GRADE system	High: 6 Moderate: 11 Low: 18 Very low quality: 0	Strong recommendation: 12 Weak recommendation: 8
Gupta et al., 2020 [42]	GRADE system	High quality: 0 Moderate quality: 2 Low quality: 3 Very low quality: 0	Strong recommendation:3 Conditional recommendation: 2
Wang et al., 2022 [44]	GRADE system	High: 11 Moderate: 15 Low: 22	Strong recommendation: 39 Weak recommendation: 9

*CPG ID* clinical practice guideline identifier

After reevaluation of the CPGs, a discrepancy was observed between the distribution of the strength of recommendations and the quality of evidence in the CPGs. Among the 235 recommendations and the corresponding evidence, 64.4% were classified as strong recommendations, whereas only 12.4% were deemed to be high-quality evidence. In addition, only 17.5% of the strong recommendations were supported by high-quality evidence. Additionally, 38.3% of the evidence was moderate quality, 48.9% was considered low quality, and 0.4% was categorized as very low quality. Notably, among all CPGs, the CPG of the Spleen and Stomach Diseases Branch of the China Association of Traditional Chinese Medicine (SSDBCATCM) has the largest proportion of strong recommendations and one of the largest proportions of high-level evidence in its recommendations and evidence [44]. Specifically, high-level evidence accounted for 22.9% of all its evidence, while strong recommendations accounted for 81.3% of all its recommendations. In contrast, the CPG of the Standards of Practice Committee of the American Society for Gastrointestinal Endoscopy (SPCASGE) [38] had only 3.0% high-quality evidence, and only 12.1% of its recommendations were classified as strong (Table 7).

**Table 7:** Distribution of quality of evidence and strength of recommendations among evidence-based CPGs after synthesis with the GRADE system

CPG ID	Number of recommendations	Level of Evidence, No. (%)				Strength of Recommendation, No. (%)	
		High	Moderate	Low	Very low	Strong	Weak
Tang et al., 2012 [37]	56	2 (3.6)	42 (75)	12 (21.4)	0	46 (79.3)	12 (20.7)
Sharaf et al., 2013 [38]	33	1 (3.0)	3 (9.1)	28 (84.8)	1 (3.1)	4 (12.1)	29 (87.9)
Evans et al., 2015 [39]	16	4 (25.0)	4 (25.0)	8 (50.0)	0	8 (50.0)	8 (50.0)
Banks et al., 2019 [40]	31	3 (9.7)	9 (29.0)	19 (61.3)	0	25 (80.6)	6 (19.4)
Lahner et al., 2019 [41]	11	2 (18.2)	4 (36.4)	5 (45.5)	0	6 (54.5)	5 (45.5)
Pimentel-Nunes et al., 2019 [4]	35	6 (17.1)	11 (35.5)	18 (51.4)	0	12 (34.3)	8 (22.9)
Gupta et al., 2020 [42]	5	0	2 (40.0)	3 (60.0)	0	3 (60.0)	2 (40.0)
Wang et al., 2022 [44]	48	11 (22.9)	15 (31.3)	22 (45.8)	0	39 (81.3)	9 (18.7)
Total, No. (%)	235	29 (12.3)	90 (38.3)	115 (48.9)	1 (0.0)	143 (60.9)	79 (33.6)

*CPG ID* clinical practice guideline identifier, *GRADE* Grading of Recommendation Assessment, Development, and Evaluation

## Discussion

The overall quality of the methodology, recommendations, and reporting of the CPGs for GPL was low. Furthermore, only one of the nine CPGs was considered high quality. According to the evaluation results, the quality of the CPGs was highly heterogeneous, and the same CPGs had different scores in different domains. There was a significant correlation between the scores on some AGREE II, RIGHT, and AGREE-REX domains. Although the eight included CPGs were deemed evidence-based, the strong recommendations put forth by these CPGs were generally not supported by high-quality evidence.

After applying the screening criteria to identify relevant CPGs, nine qualified CPGs were identified, with only the CPG of the British Society of Gastroenterology [40] deemed of high quality. CPGs aim to assist doctors and patients in making informed healthcare decisions in specific clinical scenarios, ultimately enhancing the quality of care provided to patients [45]; however, low-quality CPGs’ invalid care recommendations may be confused by users [18]. Furthermore, the nine identified CPGs originated from five different countries or regions; however, owing to the complexity and changing global healthcare landscape [46], and this limited source may not fully address the challenge. Broader sources of CPGs will allow more professional medical experts from different countries and regions will be able to participate and share their experiences and perspectives to better adapt to the different medical needs of different regions.

Nine CPGs demonstrated a clear overall purpose and scope with concise expressions. However, the quality of domain 3 (rigour of development) was not sufficiently high and there is room for improvement. CPGs must rely on reliable evidence, and recommendations must be adopted to be truly beneficial [28]. Moreover, there is a need to clearly communicate the methodology used to make recommendations so that health professionals can understand the evidence on which the clinical recommendations they are considering implementing are based [47]. Among the six domains, domain 2 (stakeholder involvement) performed poorly, with item 5 (the patients’ views and preferences have been sought) having the lowest mean score among all items. Addressing patient and stakeholder engagement is often challenging, potentially impeding optimal CPG development and affecting future patient engagement [48]. However, there is a growing recognition of the importance of incorporating the perspectives of patient groups to which CPGs are applicable, and it is crucial to address these perspectives and involve stakeholders in future recommendations [49, 50]. Domain 5 (applicability) had the lowest mean score of all domains. This is a crucial domain, as not all healthcare settings can fulfill the checklist and may need to adapt recommendations to deliver care [51]. Consequently, a lack of applicability may affect the actual implementation of CPGs, which in turn may negatively affect patient outcomes and experiences [52]. Given the diversity of clinical practice and wide use of CPGs, it is critical to analyze potential barriers to recommendations in CPGs [53].

Among the seven RIGHT domains, the basic information domain had the best mean reporting rate, which could effectively provide basic information to help target users better understand the CPGs. It is worth noting that certain CPGs may encounter potential interference from external interests, consequently jeopardizing their objectivity and reliability and diminishing their overall quality [54]. Consequently, the domains of funding, benefit statements, and management performed the worst and were unfavorable for the quality of the CPGs. Developers may need to scrutinize CPGs more closely for potential conflicts of interest to maintain objectivity in medical practice. The absence of reporting on Item 8b in all CPGs suggests a lack of specific recommendations or details regarding the execution and implementation of CPGs. This is detrimental to helping clinicians implement CPGs and may have a negative impact on medical practice. To ensure effective implementation and assessment of implementation outcomes, enhancing the reporting rate in this domain for CPGs that require enforcement and implementation is advisable.

Among the three domains of the AGREE-REX, domain values and preferences had the lowest mean scores, with the three items displaying exceptionally low scores falling under this domain. Regarding values and preferences, a systematic review evaluating the incorporation of patient perspectives in the development of CPGs found that, while most institutions advocate for the inclusion of patients and their perspectives [55], there is a shortage of specific information on the methods employed to achieve this, which is also present in the CPGs we included. This can lead to CPG recommendations with insufficient consideration of patient needs, compromising patient experience. Therefore, CPG authors need to pay more attention to values and preferences, how to effectively incorporate patients’ views, further strengthen details, and consider more diverse needs.

After considering the evaluation results, we found that the overall scores of AGREE II and RIGHT in these two CPGs [4, 40] were the highest, and the scores in various domains of each tool were relatively high, and the scores in the domain of applicability were also high. Therefore, we believe that these two CPGs [4, 40] are the most suitable for clinicians among the CPGs we evaluated.

In the subgroup analysis of the scores of AGREE II, RIGHT, and AGREE-REX domains, we discovered that the declaration of member conflicts of interest during CPG development affected the scores of both the AGREE II editorial independence domain and the RIGHT “Funding and declaration and management of interests” domain. To ensure that external influences do not diminish the quality of CPGs, we recommend disclosing conflicts of interest among members as widely as possible during future CPG development. Our study also revealed that the publication time affects the RIGHT evaluation score. Newer CPGs had higher scores than older CPGs, indicating that CPGs enhanced reporting practices over time. In addition, the correlation analysis conducted on the AGREE II, RIGHT, and AGREE-REX domains indicated associations between some aspects of methodology, recommendations, and reporting quality.

Several CPGs used the GRADE system to develop recommendations, which helped provide consistent evidence evaluation and recommendations. Nevertheless, disparities were present in the dispersion of evidence quality and recommendation strength among different CPGs. For instance, the SSDBCATCM CPG [44] had stronger recommendations, whereas the SPCASGE CPG [38] had many weak recommendations. Among the CPGs examined, 235 recommendations were identified. Of these, 143 (64.4%) were classified as strong, whereas only 25 (17.5%) were supported by high-quality evidence. High-quality evidence is scarce, and this discrepancy in the proportions is noteworthy. We feel that this inconsistency may be related to the lack of rigor in the development of CPGs, which may be reflected in the scores of domain 3 of AGREE II (Rigour of development), so it may be of interest to investigate the correlation between the scores of domain 3 of AGREE II and the degree of inconsistency. Given the small number of CPGs included in our study, we plan to include more CPGs in the future to explore this association and to explore the reasons for the inconsistency between the strength of recommendations and the level of evidence. Besides, for some special patients, such as the elderly, children, pregnant women, patients with rare diseases, or responding to health emergencies, although the level of evidence is low, it still may provides important and effective clinical information, so developers of CPGs may give strong recommendations [56]. In addition, degree of some recommendations may be defined as strong even if they are not supported by high-level research evidence because of good observed clinical effects. However, it is important to note that strong recommendations necessitate a favorable balance between benefits, harms, and burdens, a criterion that is seldom fulfilled [56, 57]. Consequently, strong recommendations based on low-quality evidence are rarely appropriate. Expressing strong recommendations for recommendations that do not have high-quality evidence may lead healthcare practitioners to overemphasize certain treatments that do not work as expected. Therefore, in addition to focusing on the quality of the CPG domains, the production process of subsequent CPGs should consider the consistency of strong recommendations and high-quality evidence.

Three assessment tools were used to assess the methodological quality, recommendation quality, and reporting quality and to analyze the evidence and strength of CPG recommendations for GPL. Through a multidimensional comprehensive evaluation, we found some deficiencies in the development of current CPGs and identified room for improvement in the development of future CPGs. It can help improve the development process and reporting content for future CPGs, optimize CPG recommendations, and continuously improve the quality of CPGs. Moreover, the introduction of rigorously evaluated CPGs can help improve clinical practice [58].

A notable strength of this study lies in its utilization of the AGREE II, RIGHT, and AGREE-REX tools for evaluating CPGs. Incorporating multiple assessment tools allows for a more comprehensive examination, facilitating the identification of areas for improvement within CPGs. Moreover, the advantage of using multiple assessment tools is that they can check whether the aspects of concern of the CPG development team have received sufficient attention, thus helping to better grasp the full picture of CPG development. Simultaneously, the recommendations and evidence in the CPGs were analyzed, focusing on the consistency between the strength of the recommendations and the quality of evidence.

Regarding constraints, despite our comprehensive literature search of the database, it is possible that certain CPGs were not identified, and there may have been some CPGs that met the inclusion criteria but were inadvertently overlooked. Furthermore, our study was limited to CPGs published in English, which restricted the number of CPGs included in the analysis. Additionally, although AGREE II, RIGHT, and AGREE-REX can be employed to evaluate the methodology, reporting, and recommendation quality of CPGs, these assessment tools do not offer an exhaustive examination of the content within the CPGs. The sample size of this study was small, only 9 CPGs, which may lead to high randomness and low external validity of the results of subgroup analysis. The inclusion of only English literature should be an important reason for the small sample size. However, the results of this study still have certain reference significance for the development and research of related CPGs in the future.

## Conclusion

Through this evaluation of the CPGs for GPL, we found that there are many deficiencies in the quality of the current CPGs for GPL, and the overall quality was low. The methodology, reporting, and recommendation quality of CPGs for GPL needs to be further improved. These two CPGs [4, 40] are the ones we think are the most applicable to clinicians. Future CPG development should adopt more rigorous and transparent criteria to ensure the creation and implementation of high-quality CPGs that effectively address the healthcare requirements of both patients and the general population. In terms of recommendations for CPGs, more attention should be paid to the consistency of the strength of recommendations and the quality of evidence in developing future CPGs to ensure that the strength of recommendations is appropriate.

## Supplementary Information

Supplementary Material 1.

## Data Availability

The datasets used and/or analysed during the current study are available from the corresponding author on reasonable request.
