# Supplementary material for: Assessing the quality and integrating the evidence and strength of recommendations in the guidelines for gastric precancerous lesions
Source: BMC Cancer. 2025 Feb 15;25:272. doi: 10.1186/s12885-025-13687-y (PMC11830177; doi:10.1186/s12885-025-13687-y)
Supplement: Supplementary file 1 — Supplementary Material 1. [file 12885_2025_13687_MOESM1_ESM.zip › supplementary materials/Supplementary Table.docx]

Table 1. Overall mean (SD) scores for each AGREE II item of included CPGs.

| AGREE II item | Mean ± SD |
| --- | --- |
| 1 (The overall objective(s) of the guideline is (are) specifically described.) | 5.2±1.5 |
| 2 (The health question(s) covered by the guideline is (are) specifically  described.) | 5.4±1 |
| 3 (The population (patients, public, etc.) to whom the guideline is meant to apply is specifically described.) | 3.9±1.6 |
| 4 (The guideline development group includes individuals from all relevant professional groups.) | 4.4±1.3 |
| 5 (The views and preferences of the target population (patients, public, etc.) have been sought.) | 1.3±0.7 |
| 6 (The target users of the guideline are clearly defined.) | 3.1±2.3 |
| 7 (Systematic methods were used to search for evidence.) | 4.2±2 |
| 8 (The criteria for selecting the evidence are clearly described.) | 2.9±1.7 |
| 9 (The strengths and limitations of the body of evidence are clearly  described.) | 5.2±1.7 |
| 10 (The methods for formulating the recommendations are clearly described.) | 3.7±2 |
| 11 (The health benefits, side effects, and risks have been considered in formulating the recommendations.) | 4.3±1.6 |
| 12 (There is an explicit link between the recommendations and thesupporting evidence.) | 5.7±1.3 |
| 13 (The guideline has been externally reviewed by experts prior to its  Publication.) | 2.9±1.9 |
| 14 (A procedure for updating the guideline is provided.) | 2.9±2.2 |
| 15 (The recommendations are specific and unambiguous.) | 5.1±1 |
| 16 (The different options for management of the condition or health issue are clearly presented.) | 5.7±1.1 |
| 17 (Key recommendations are easily identifiable.) | 5.1±1.5 |
| 18 (The guideline describes facilitators and barriers to its application.) | 2.3±1.6 |
| 19 (The guideline provides advice and/or tools on how the recommendations can be put into practice.) | 2±1.5 |
| 20 (The potential resource implications of applying the recommendations have been considered.) | 1.7±1.3 |
| 21 (The guideline presents monitoring and/or auditing criteria.) | 3.9±1.7 |
| 22 (The views of the funding body have not influenced the content of the guideline.) | 2.6±2.3 |
| 23 (Competing interests of guideline development group members have been recorded and addressed.) | 3.8±2 |

CPG, clinical practice guideline; AGREE, Appraisal of Guidelines for Research and Evaluation.

Table 2. Inter-rater reliability for AGREE Il domain and overall rating.

| AGREE II domain and overall rating | ICC(95%CI) |
| --- | --- |
| Domain 1 | 0.807 (0.671,0.899) |
| Domain 2 | 0.931 (0.875,0.966) |
| Domain 3 | 0.850 (0.788,0.898) |
| Domain 4 | 0.848 (0.735,0.922) |
| Domain 5 | 0.821 (0.715,0.897) |
| Domain 6 | 0.940 (0.875,0.975) |
| Over ratting | 0.871 (0.659,0.966) |

AGREE, Appraisal of Guidelines for Research and Evaluation; ICC, intraclass correlationcoefficients; Cl, confidence interval

Table 3. Overall mean (SD) scores for each RIGHT item of included CPGs.

| RIGHT item | Mean ± SD |
| --- | --- |
| 1a (Title/subtitle: Identify the report as a guideline, that is, with "guideline(s)" or "recommendation(s)" in the title.) | 0.8±0.4 |
| 1b (Title/subtitle: Describe the year of publication of the guideline.) | 1±0 |
| 1c (Title/subtitle: Describe the focus of the guideline, such as screening, diagnosis, treatment, management, prevention, or others.) | 0.7±0.4 |
| 2 (Executive summary) | 0.8±0.3 |
| 3 (Abbreviations and acronyms) | 0.6±0.3 |
| 4 (Corresponding developer) | 0.6±0.5 |
| 5 (Brief description of the health problem(s)) | 0.6±0.4 |
| 6 (Aim(s) of the guideline and specific objectives) | 0.9±0.3 |
| 7a (Target population(s): Describe the primary population(s) that is affected by the recommendation(s) in the guideline) | 0.6±0.3 |
| 7b (Target population(s): Describe any subgroups that are given special consideration in the guideline) | 0.6±0.4 |
| 8a (End users and settings: Describe the intended primary users of the guideline (such as primary care providers, clinical specialists, public health practitioners, program managers, and policymakers) and other potential users of the guideline) | 0.6±0.4 |
| 8b (End users and settings: Describe the setting(s) for which the guideline is intended, such as primary care, low- and middle-income countries, or inpatient facilitiesDescribe the setting(s) for which the guideline is intended, such as primary care, low- and middle-income countries, or inpatient facilities) | 0±0 |
| 9a (Guideline development groups: Describe how all contributors to the guideline development were selected and their roles and responsibilities (e.g., steering group, guideline panel, external reviewers, systematic review team, and methodologists)) | 0.4±0.3 |
| 9b (Guideline development groups: List all individuals involved in developing the guideline, including their title, role(s), and institutional affiliation(s)) | 0.5±0.4 |
| 10a (Health care questions: State the key questions that were the basis for the recommendations in PICO (population, intervention, comparator, and outcome) or other format as appropriate) | 0.7±0.3 |
| 10b (Health care questions: Indicate how the outcomes were selected and sorted) | 0.8±0.3 |
| 11a (Systematic reviews: Indicate whether the guideline is based on new systematic reviews done specifically for this guideline or whether existing systematic reviews were used) | 0.6±0.3 |
| 11b (Systematic reviews: If the guideline developers used existing systematic reviews, reference these and describe how those reviews were identified and assessed (provide the search strategies and the selection criteria, and describe how the risk of bias was evaluated) and whether they were updated) | 0.4±0.3 |
| 12 (Assessment of the certainty of the body of evidence) | 0.9±0.3 |
| 13a (Recommendations: Provide clear, precise, and actionable recommendations) | 0.9±0.2 |
| 13b (Recommendations: Present separate recommendations for important subgroups if the evidence suggests that there are important differences in factors influencing recommendations, particularly the balance of benefits and harms across subgroups) | 0.6±0.4 |
| 13c (Recommendations: Indicate the strength of recommendations and the certainty of the supporting evidence) | 0.9±0.3 |
| 14a (Rationale/explanation for recommendations: Describe whether values and preferences of the target population(s) were considered in the formulation of each recommendation. If yes, describe the approaches and methods used to elicit or identify these values and preferences. If values and preferences were not considered, provide an explanation) | 0.4±0.2 |
| 14b (Rationale/explanation for recommendations: Describe whether cost and resource implications were considered in the formulation of recommendations. If yes, describe the specific approaches and methods used (such as cost-effectiveness analysis) and summarize the results. If resource issues were not considered, provide an explanation) | 0.2±0.3 |
| 14c (Rationale/explanation for recommendations: Describe other factors taken into consideration when formulating the recommendations, such as equity, feasibility, and acceptability) | 0.3±0.3 |
| 15 (Evidence to decision processes) | 0.9±0.2 |
| 16 (External review) | 0.5±0.5 |
| 17 (Quality assurance) | 0.6±0.2 |
| 18a (Funding source(s) and role(s) of the funder: Describe the specific sources of funding for all stages of guideline development) | 0.3±0.4 |
| 18b (Funding source(s) and role(s) of the funder: Describe the role of funder(s) in the different stages of guideline development and in the dissemination and implementation of the recommendations) | 0.3±0.4 |
| 19a (Declaration and management of interests: Describe what types of conflicts (financial and nonfinancial) were relevant to guideline development) | 0.4±0.4 |
| 19b (Declaration and management of interests: Describe how conflicts of interest were evaluated and managed and how users of the guideline can access the declarations) | 0.5±0.4 |
| 20 (Access) | 0.6±0.3 |
| 21 (Suggestions for further research) | 0.6±0.3 |
| 22 (Limitations of the guideline) | 0.6±0.3 |

CPG, clinical practice guideline; RIGHT: Reporting Items for Practice Guidelines in Healthcare.

Table 4. Overall mean (SD) scores for each AGREE-REX item of included CPGs.

| AGREE-REX item | Mean ± SD |
| --- | --- |
| 1 (Evidence) | 3.1±1.3 |
| 2 (Applicability target users) | 3.6±1.1 |
| 3 (Applicability to patients/ population) | 2.1±0.9 |
| 4 (Values and preferences of target users) | 1.4±0.5 |
| 5 (Values and preferences of patients/population) | 1.8±1.2 |
| 6 (Values and preferences of policy/decision makers) | 1.1±0.3 |
| 7 (Values and preferences of guideline developers) | 1.3±0.7 |
| 8 (Purpose) | 3.2±1 |
| 9 (Local application and adoption) | 1.7±0.5 |

CPG, clinical practice guideline; AGREE-REX: Appraisal of Guidelines for Research and Evaluation-Recommendation Excellence.
