# Supplementary material for: Assessing the quality and integrating the evidence and strength of recommendations in the guidelines for gastric precancerous lesions
Source: BMC Cancer. 2025 Feb 15;25:272. doi: 10.1186/s12885-025-13687-y (PMC11830177; doi:10.1186/s12885-025-13687-y)
Supplement: Supplementary file 1 — Supplementary Material 1. [file 12885_2025_13687_MOESM1_ESM.zip › supplementary materials/Supplementary File 1.doc]

#### **PubMed Search Strategy**

#1 "Guidelines as Topic"[Mesh]

#2 "Guideline" [Publication Type]

#3 guideline*[Title/Abstract] OR consensus[Title/Abstract] OR recommendation*[Title/Abstract] OR guidance[Title/Abstract] OR standard[Title] OR statement*[Title/Abstract]

#4 #1 OR #2 OR #3

#5 "Gastritis, Atrophic"[Mesh]

#6 Atrophic Gastritides[Title/Abstract] OR Atrophic Gastritis[Title/Abstract] OR Gastritides, Atrophic[Title/Abstract]

#7 #5 OR #6

#8 gastric dysplasia[Title/Abstract] OR intestinal metaplasia[Title/Abstract] OR chronic atrophic gastritis[Title/Abstract] OR Autoimmune gastritis[Title/Abstract] OR Gastric intestinal metaplasia[Title/Abstract] OR gastric polyp[Title/Abstract] OR Gastric adenoma[Title/Abstract] OR Gastric precancerous lesion[Title/Abstract] OR Precancerous lesion of gastric cancer[Title/Abstract]

#9	"Gastritis, Hypertrophic"[Mesh]

#10	Hypertrophic Gastritis[Title/Abstract] OR Menetrier Disease[Title/Abstract] OR Disease, Menetrier[Title/Abstract] OR Menetrier's Disease[Title/Abstract] OR Disease, Menetrier's[Title/Abstract] OR Menetriers Disease[Title/Abstract] OR Giant Hypertrophic Gastritis[Title/Abstract] OR Gastritis, Giant Hypertrophic[Title/Abstract] OR Hypertrophic Gastritis, Giant[Title/Abstract] OR Gastritis, Familial Giant Hypertrophic[Title/Abstract]

#11 #9 OR #10

#12 #7 AND #4

#13 #8 AND #4

#14 #11 AND #4

#15 #12 OR #13 OR #14

#16 #15 Filters: from 2011/1/1 - 2023/4/1

Results: 1055

##### [**Embase**](https://www.embase.com/) **Search Strategy**

#1 'guidelines as topic'/exp

#2 guideline:it

#3 guideline:ab,ti OR consensus:ab,ti OR recommendation:ab,ti OR guidance:ab,ti OR standard:ab,ti OR statement:ab,ti

#4 #1 OR #2 OR #3

#5 'intestinal metaplasia'/exp

#6  'intestine metaplasia':ab,ti

#7 #5 OR #6

#8 'atrophic gastritis'/exp

#9 'autoimmune gastritis':ab,ti OR 'chronic atrophic gastritis':ab,ti OR 'gastric atrophy':ab,ti OR 'gastritis, atrophic':ab,ti

#10 #8 OR #9

#11 'intestinal metaplasia of the stomach'/exp

#12 'gastric cardia intestinal metaplasia':ab,ti OR 'gastric cardiac intestinal metaplasia':ab,ti OR 'gastric intestinal metaplasia':ab,ti OR 'intestinal metaplasia in human stomach':ab,ti OR 'intestinal metaplasia of the gastric cardia':ab,ti OR 'intestinal metaplasia of the gastric mucosa':ab,ti

#13 #11 OR #12

#14 'gastric dysplasia':ab,ti OR 'gastric adenoma':ab,ti OR 'gastric precancerous lesion':ab,ti OR 'precancerous lesion of gastric cancer':ab,ti

#15 'menetrier disease'/exp

#16 'benign giant hypertrophic gastritis':ab,ti OR 'disease, menetrier':ab,ti OR 'gastritis, benign giant hypertrophic':ab,ti OR 'gastritis, hypertrophic':ab,ti OR 'giant hypertrophic gastritis':ab,ti OR 'hypertrophic gastritis':ab,ti OR 'hypertrophic gastritis, giant, benign':ab,ti OR 'hypertrophic gastropathy':ab,ti OR 'menetriers disease':ab,ti OR 'stomach menetrier disease':ab,ti

#17 #15 OR #16

#18 'gastric stump':ab,ti OR 'gastric stumps':ab,ti OR 'stump, gastric':ab,ti OR 'stumps, gastric':ab,ti OR 'residual stomach':ab,ti OR 'residual stomachs':ab,ti OR 'stomach, residual':ab,ti OR 'stomachs, residual':ab,ti OR 'remnant stomach':ab,ti OR 'remnant stomachs':ab,ti OR 'stomach, remnant':ab,ti OR 'stomachs, remnant':ab,ti OR 'gastric remnant':ab,ti OR 'gastric remnants':ab,ti OR 'remnant, gastric':ab,ti OR 'remnants, gastric':ab,ti

#19 #7 AND #4

#20 #10 AND #4

#21 #13AND #4

#22 #14AND #4

#23 #17 AND #4

#24 #18 AND #4

#25 #19 OR #20 0R #21 OR #22 OR #23 OR #24

#26 #25 AND [2011-2023]/py

Results: 1399

#### **Cochrane library** **Search Strategy**

#1 MeSH descriptor: [Guidelines as Topic] explode all trees

#2 "guideline":pt

#3 (guideline):ti,ab,kw OR (consensus):ti,ab,kw OR (recommendation):ti,ab,kw OR (guidance):ti,ab,kw OR (standard):ti,ab,kw OR (statement):ti,ab,kw

#4 #1 OR #2 OR #3

#5 MeSH descriptor: [Gastritis, Atrophic] explode all trees

#6 (Atrophic Gastritides):ti,ab,kw OR (Atrophic Gastritis):ti,ab,kw OR (Gastritides, Atrophic):ti,ab,kw

#7 #5 OR #6

#8 (gastric dysplasia):ti,ab,kw OR (intestinal metaplasia):ti,ab,kw OR (chronic atrophic gastritis):ti,ab,kw OR (Autoimmune gastritis):ti,ab,kw OR (Gastric intestinal metaplasia):ti,ab,kw OR (gastric polyp):ti,ab,kw OR (Gastric adenoma):ti,ab,kw OR (Gastric precancerous lesion):ti,ab,kw OR (Precancerous lesion of gastric cancer):ti,ab,kw

#9 MeSH descriptor: [Gastric Stump] explode all trees

#10 (Gastric Stumps):ti,ab,kw OR (Stump, Gastric):ti,ab,kw OR (Residual Stomachs):ti,ab,kw OR (Gastric Remnant):ti,ab,kw OR (Stomach, Remnant):ti,ab,kw OR (Stomachs, Remnant):ti,ab,kw OR (Remnant, Gastric):ti,ab,kw OR (Stumps, Gastric):ti,ab,kw OR (Gastric Remnants):ti,ab,kw OR (Remnants, Gastric):ti,ab,kw OR (Remnant Stomachs):ti,ab,kw OR (Stomachs, Residual):ti,ab,kw OR (Remnant Stomach):ti,ab,kw OR (Stomach, Residual):ti,ab,kw OR (Residual Stomach):ti,ab,kw

#11 #9 OR #10

#12 MeSH descriptor: [Gastritis, Hypertrophic] explode all trees

#13 (Hypertrophic Gastritis, Giant):ti,ab,kw OR (Disease, Menetrier):ti,ab,kw OR (Gastritis, Giant Hypertrophic):ti,ab,kw OR (Giant Hypertrophic Gastritis):ti,ab,kw OR (Disease, Menetrier's):ti,ab,kw OR (Menetrier Disease):ti,ab,kw OR (Menetrier's Disease):ti,ab,kw OR (Menetriers Disease):ti,ab,kw OR (Hypertrophic Gastritis):ti,ab,kw OR (Gastritis, Familial Giant Hypertrophic):ti,ab,kw

#14 #12 OR #13

#15 #4 AND #7

#16 #4 AND #8

#17 #4 AND #11

#18 #4 AND #14

#19 #15 OR #16 OR #17 OR #18

#20 #19 with Cochrane Library publication date from Jan 2011 to Apr 2023

Results: 225

#### **CINAHL Search Strategy**

S1 SU Guidelines as Topic

S2  PT Guideline

S3 TX guideline OR TX consensus OR TX recommendation OR TX guidance OR TX standard OR TX statement

S4 S1 OR S2 OR S3

S5 SU Gastritis, Atrophic

S6 TI Atrophic Gastritides OR TI Atrophic Gastritis OR TI Gastritides, Atrophic

S7 S5 OR S6

S8 TI gastric dysplasia OR TI intestinal metaplasia OR TI chronic atrophic gastritis OR TI Autoimmune gastritis OR TI Gastric intestinal metaplasia OR TI gastric polyp OR TI Gastric adenoma OR TI Gastric precancerous lesion OR TI Precancerous lesion of gastric cancer

S9 SU Gastric Stump

S10 TI Gastric Stumps OR TI Stump, Gastric OR TI Stumps, Gastric OR TI Residual Stomach OR TI Residual Stomachs OR TI Stomach, Residual OR TI Stomachs, Residual OR TI Remnant Stomach OR TI Remnant Stomachs OR TI Stomach, Remnant OR TI Stomachs, Remnant OR TI Gastric Remnant OR TI Gastric Remnants OR TI Remnant, Gastric OR TI Remnants, Gastric

S11 S9 OR S10

S12 SU Gastritis, Hypertrophic

S13  TI Hypertrophic Gastritis OR TI Menetrier Disease OR TI Disease, Menetrier OR TI Menetrier's Disease OR TI Disease, Menetrier's OR TI Menetriers Disease OR TI Giant Hypertrophic Gastritis OR TI Gastritis, Giant Hypertrophic OR TI Hypertrophic Gastritis, Giant OR TI Gastritis, Familial Giant Hypertrophic

S14 S12 OR S13

S15 S4 AND S7

S16 S4 AND S8

S17 S4 AND S11

S18 S4 AND S14

S19 S15 OR S16 OR S17 OR S18

S20 S19 Published Date: 20110101-20230401

Results: 86

#### NICE (2)

gastric dysplasia (2)

#### SIGN (0)

#### GIN (0)

#### AHRQ (1)

Gastritis, Atrophic (1)

#### NHMRC (0)

#### WHO (0)
